# α-Synucleinopathy associated with G51D *SNCA* mutation: a link between Parkinson’s disease and multiple system atrophy?

**DOI:** 10.1007/s00401-013-1096-7

**Published:** 2013-02-12

**Authors:** Aoife P. Kiely, Yasmine T. Asi, Eleanna Kara, Patricia Limousin, Helen Ling, Patrick Lewis, Christos Proukakis, Niall Quinn, Andrew J. Lees, John Hardy, Tamas Revesz, Henry Houlden, Janice L. Holton

**Affiliations:** 1 Queen Square Brain Bank, UCL Institute of Neurology, London, UK; 2 Department of Molecular Neuroscience, UCL Institute of Neurology, Queen Square, London, UK; 3 Department of Molecular Neuroscience, UCL Institute of Neurology, London, UK; 4 Reta Lila Weston Institute of Neurological Studies, UCL Institute of Neurology, London, UK; 5 Unit of Functional Neurosurgery, UCL Institute of Neurology, London, UK; 6 Sobell Department of Motor Neuroscience and Movement Disorders, University College London, London, UK; 7 School of Pharmacy, University of Reading, Whiteknights, Reading, UK; 8 Department of Clinical Neuroscience, UCL Institute of Neurology, London, UK; 9 National Hospital for Neurology and Neurosurgery, Queen Square, London, UK

**Keywords:** Parkinson’s disease, Multiple system atrophy, α-Synuclein, SNCA

## Abstract

We report a British family with young-onset Parkinson’s disease (PD) and a G51D *SNCA* mutation that segregates with the disease. Family history was consistent with autosomal dominant inheritance as both the father and sister of the proband developed levodopa-responsive parkinsonism with onset in their late thirties. Clinical features show similarity to those seen in families with *SNCA* triplication and to cases of A53T *SNCA* mutation. Post-mortem brain examination of the proband revealed atrophy affecting frontal and temporal lobes in addition to the caudate, putamen, globus pallidus and amygdala. There was severe loss of pigmentation in the substantia nigra and pallor of the locus coeruleus. Neuronal loss was most marked in frontal and temporal cortices, hippocampal CA2/3 subregions, substantia nigra, locus coeruleus and dorsal motor nucleus of the vagus. The cellular pathology included widespread and frequent neuronal α-synuclein immunoreactive inclusions of variable morphology and oligodendroglial inclusions similar to the glial cytoplasmic inclusions of multiple system atrophy (MSA). Both inclusion types were ubiquitin and p62 positive and were labelled with phosphorylation-dependent anti-α-synuclein antibodies In addition, TDP-43 immunoreactive inclusions were observed in limbic regions and in the striatum. Together the data show clinical and neuropathological similarities to both the A53T *SNCA* mutation and multiplication cases. The cellular neuropathological features of this case share some characteristics of both PD and MSA with additional unique striatal and neocortical pathology. Greater understanding of the disease mechanism underlying the G51D mutation could aid in understanding of α-synuclein biology and its impact on disease phenotype.

**Electronic supplementary material:**

The online version of this article (doi:10.1007/s00401-013-1096-7) contains supplementary material, which is available to authorized users.

## Introduction

The term α-synucleinopathy unites a group of neurodegenerative diseases which share the pathological hallmark of fibrillary inclusions in which α-synuclein protein is the major component. The three most common members of this group are Parkinson’s disease (PD), dementia with Lewy bodies (DLB) and multiple system atrophy (MSA). PD and DLB have common neuropathological features including deposition of fibrillar α-synuclein in Lewy bodies (LB) and Lewy neurites (LN). In MSA, α-synuclein is aggregated in oligodendrocytes forming the hallmark lesion, the glial cytoplasmic inclusion (GCI), and also in neuronal cytoplasmic inclusions (NCIs), cell processes and to a lesser extent in neuronal and glial nuclei [1, 28].

Several missense mutations of the *SNCA* gene have been identified in families with autosomal dominant forms of PD. No *SNCA* mutation has been linked with MSA, however, polymorphisms of the gene have been associated with increased risk of the disease in Caucasian populations, although these results have not been replicated in all populations [2, 70, 71, 83]. The *SNCA* gene, which encodes the 140-amino acid protein α-synuclein, is located on chromosome 4q21-23. In Parkinson’s disease, the first *SNCA* mutation to be described was A53T in a Greek-Italian family [62] and this was subsequently identified in families of Asian, Swedish and Polish origin [5, 12, 42, 49, 59, 61, 66, 77]. Two further missense mutations of *SNCA* were identified, A30P [43] and E46K [84] in German and Basque families, respectively. Very recently, we have reported the novel H50Q *SNCA* mutation [65]. Missense mutations in the N-terminal region of α-synuclein are reported to have a direct impact on α-synuclein conformation and function. The A53T and E46K mutant forms of α-synuclein exhibit faster fibrillisation kinetics than wild-type protein [12], while fibrillisation of the A30P mutant protein is slower and results in fewer complex fibrils in LBs [47]. Duplication and triplication of *SNCA* have been discovered in a small number of families and sporadic cases of levodopa-responsive PD and, where described, pathological features of PD, in addition to GCIs similar to those of MSA, are observed [10, 22, 27, 31, 32, 55, 56]. The number of *SNCA* locus replicates is known to influence disease progression, such that triplication causes earlier onset and a more rapid clinical course than *SNCA* duplication [31, 75].

Pathological inclusions of fibrillar α-synuclein have distinct morphologies and distribution depending on disease type. For example, in PD, the characteristic LBs and LNs occur in brainstem nuclei and usually exhibit a hierarchical spread to involve limbic and neocortical regions with disease progression [8], although not all cases conform to the proposed pattern of disease progression [36]. In MSA, GCIs are the most abundant form of fibrillar α-synuclein inclusion and together with neuronal cytoplasmic or nuclear inclusions are distributed widely in the striatonigral, olivopontocerebellar and other regions [1, 4, 33, 60].

We report a family with young-onset PD and a mutation in *SNCA* that segregates with the disease. We describe an α-synucleinopathy with both PD and MSA-like neuropathological features together with involvement of the striatum and severe CA2/3 neuronal loss. The distribution of neuronal and oligodendroglial inclusions immunoreactive for α-synuclein, ubiquitin and p62 is described. The phosphorylation state of α-synuclein within inclusions and the relationship of α-synuclein to intracellular accumulation of tau and TDP-43 are also investigated. Together the evidence reveals neuropathological similarities to both the A53T *SNCA* mutation and multiplication cases with additional unique striatal and neocortical pathology [27, 48].

## Materials and methods

### Brain tissue

The brain was donated to the Queen Square Brain Bank for Neurological Disorders, UCL Institute of Neurology using ethically approved protocols and stored for research under a licence issued by the Human Tissue Authority (No. 12198). Following fixation in 10 % buffered formalin, the right half brain was sliced in the coronal plane, examined and blocks were selected for paraffin wax embedding and histology.

Paraffin-embedded sections (8 μm) were stained using haematoxylin and eosin (H&E), Luxol fast blue/cresyl violet and Gallyas silver impregnation. Immunohistochemistry was performed as previously described [58] using primary antibodies detailed in Online Resource 1. Double immunofluorescence was detected using isotype specific anti-rabbit IgG or anti-mouse IgG secondary antibodies conjugated with either Alexa 488 or 594 fluorescent dyes (1:400) (Life technologies, Paisley, UK) followed by quenching of autofluorescence with 0.1 % Sudan Black/70 % ethanol (Sigma-Aldrich, Dorset, UK) solution for 10 min and mounting with glass coverslips using VECTAshield mounting media with 4′,6-diamidino-2-phenylindole (DAPI) nuclear stain (Vector laboratories, Peterborough, UK). Images were visualised using confocal fluorescence microscopy (Leica DM5500 B).

### Genetics

Sanger sequencing was performed for exon 3 of *SNCA* on family members for whom a DNA sample was available (affected sibling and unaffected mother). Genomic DNA was amplified through polymerase chain reaction (PCR) with the Roche Fast start master mix kit (primer sequences available upon request). After purification, the PCR product was sequenced bi-directionally with the BigDye Terminator v3.1 Cycle Sequencing Kit (Applied biosystems). Purified sequencing reaction products were run on ABI 3730xl DNA analyser, sequencing results were analysed on Sequencher version 4.1.4 and variants identified were named based on the GenBank reference sequence with accession numbers NM_001146055.1 and NP_001139527.1. Variants identified were verified through re-sequencing a duplicate genomic DNA sample. To exclude larger genomic rearrangements in the *SNCA* region, genomic DNA samples from both affected siblings were run on Illumina Human660W-Quad BeadChip arrays and the results were analysed on Illumina Genome studio V2010.2.

### Sequence alignment

Amino acid sequence data for α-synuclein from *Homo* *sapiens*, *Pan troglodytes*, *Sus scrofa*, *Mus musculus*, *Bos taurus*, *Xenopus laevis* and *Gallus gallus*, along with amino acid sequence data for human β and γ synuclein, were downloaded from NCBI. Sequence alignment was carried out using the basic local alignment search tool (BLAST, http://blast.ncbi.nlm.nih.gov). Secondary structure data for α-synuclein are based upon data from Ulmer and coworkers [80].

## Results

### Case history

At age 19, this British man presented with stiffness and tremor in his left hand. Examination revealed a classical parkinsonian rest tremor of the left arm and leg with bradykinesia, cogwheel rigidity and reduced left arm swing on walking. Eye movements were normal. His symptoms gradually involved his right side and he had a parkinsonian gait. At age 25, levodopa therapy was started with a good initial response, but 7 months later, he developed levodopa-induced choreiform movements which continued to be troublesome for a decade. Since age 28, he had progressive cognitive impairment with a mini mental state examination (MMSE) score of 24/30 at age 30 and 19/30 at age 32, neuropsychometry showed visual and verbal memory impairments and he later developed visual hallucination. At age 33, he was incapacitated by severe dysarthria, severe akinetic rigidity, postural hypotension with syncope and postural instability. Examination revealed limb myoclonus, spasticity and bilateral extensor plantar response. He started to have seizures in the last 8 years of his life but these were controlled using sodium valproate. He died at age 49 with a disease duration of 29 years.

His father died of a similar illness with onset of motor symptoms at age 39 and later developed dementia and died of sepsis at age 47. His sister developed parkinsonism at age 40 and has had a good persistent levodopa response. She is now 48 years old and has mild peak dose dyskinesia, occasional visual hallucination and has not noted any cognitive impairment. The case history of the proband was previously published in a series on young onset Parkinson’s disease (case 2M) [67].

### Genetics

**Fig. 1:**
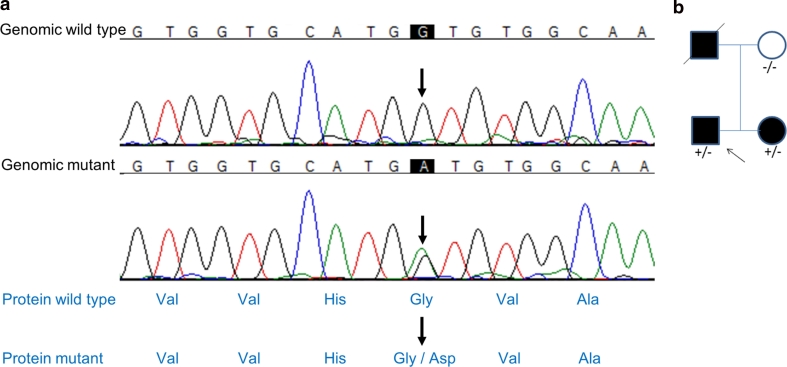
*SNCA* mutation G51D. Chromatogram (*red* thymine, *blue* cytosine, *green* adenine and *black* guanine). Sanger sequencing of the SNCA gene identified a c.G152A mutation (*upper arrows*) in exon 3 of this gene as shown. This created a glycine to aspartic acid amino acid change (*lower arrows*)

**Fig. 2:**
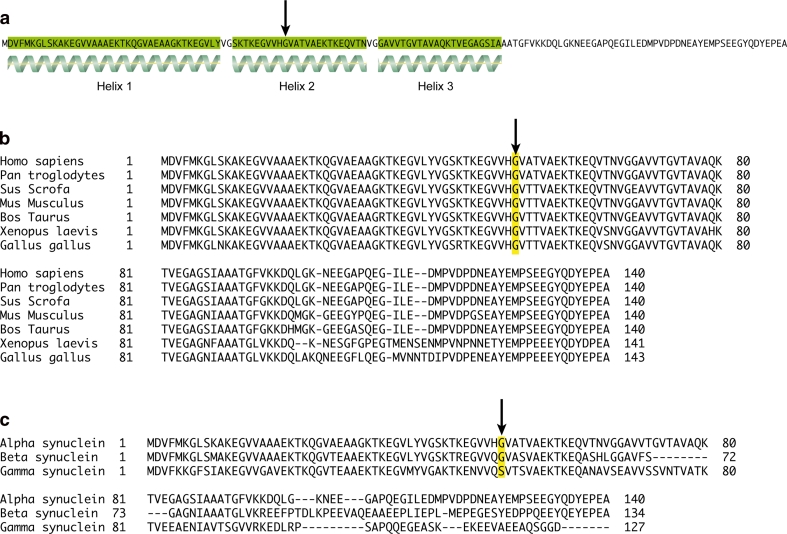
α-Synuclein protein structure and conservation as shown by amino acid sequence alignment. Amino acid sequence of human α-synuclein, showing regions of secondary structure (alpha helices highlighted in *green*). The G51 residue is indicated by an *arrow*, and sits in the middle of helix 2 **(a**). Sequence alignment of α-synuclein amino acid sequences from *Homo sapiens*, *Pan troglodytes*, *Sus scrofa*, *Mus musculus*, *Bos taurus*, *Xenopus laevis* and *Gallus gallus*. The G51 residue highlighted in *yellow* and indicated by an *arrow* is conserved throughout these organisms (**b**). Sequence alignment of human α-, β- and γ-synuclein amino acid sequences, with the G51 residue is highlighted in *yellow* and indicated by an *arrow* (**c**). This residue is conserved in α- and β-synuclein, but is replaced by a serine residue in γ-synuclein

In the affected family members, an α-synuclein G > A heterozygous mutation at base 152, codon 51 causing a glycine to aspartic acid amino acid change (Fig. 1a). This mutation segregated with the disease in the family (Fig. 1b) and was not seen in over 4,500 control individuals. Multiplication of the α-synuclein gene had been previously excluded by SNP array analysis. The mutated amino acid is highly conserved across species down to invertebrates and is predicted to be damaging using PolyPhen-2 and SIFT (Fig. 2).

### Neuropathological findings

**Fig. 3:**
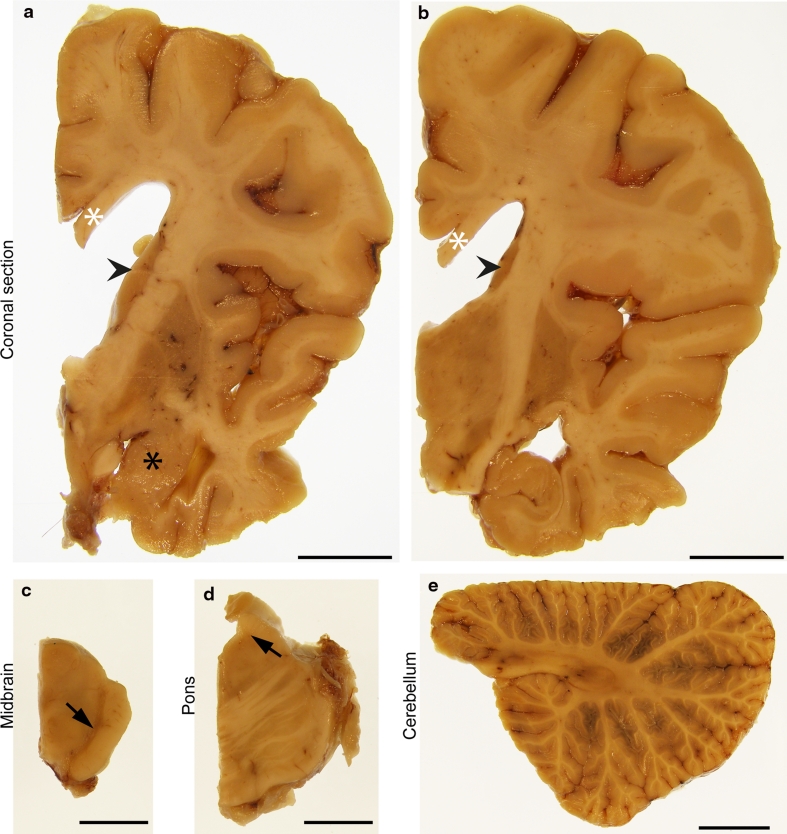
Macroscopic features. Macroscopic images of formalin fixed right hemispheric brain slices (**a**, **b**), midbrain (**c**), pons (**d**) and cerebellum (**e**). There was severe atrophy of the temporal cortex with relative preservation of the superior temporal gyrus (**a**, **b**) and severe reduction in volume of white matter with thinning of the corpus callosum (**a**, **b** *white asterisks*). The caudate nucleus was reduced in volume (**a**, **b** *arrow heads*). There was reduction in size and grey discolouration of both the putamen and the globus pallidus (**a**, **b**). The amygdala was small and darkly coloured (**a**, *black asterisk*). The hippocampus was moderately reduced in volume (**b**). The midbrain showed marked depigmentation of the substantia nigra (**c**, *arrow*). The pons was well preserved with severe pallor of the locus coeruleus (**d**, *arrow*). The cerebellum was macroscopically normal and the white matter was well preserved (**e**). *Scale bars* represent 15 mm

The brain weighed 1,036 g and showed frontal and anterior temporal lobe atrophy (Fig. 3a, b). The hippocampus, amygdala, caudate, putamen and globus pallidus were reduced in volume and showed greyish discolouration (Fig. 3a, b). The subthalamic nucleus was of normal size with mild brown discolouration (Fig. 3b). There was severe loss of pigmentation of the substantia nigra (Fig. 3c, arrow) and mild pallor of the locus coeruleus (Fig. 3d, arrow). The medulla and cerebellum were macroscopically unremarkable (Fig. 3e).

### Histological findings

**Fig. 4:**
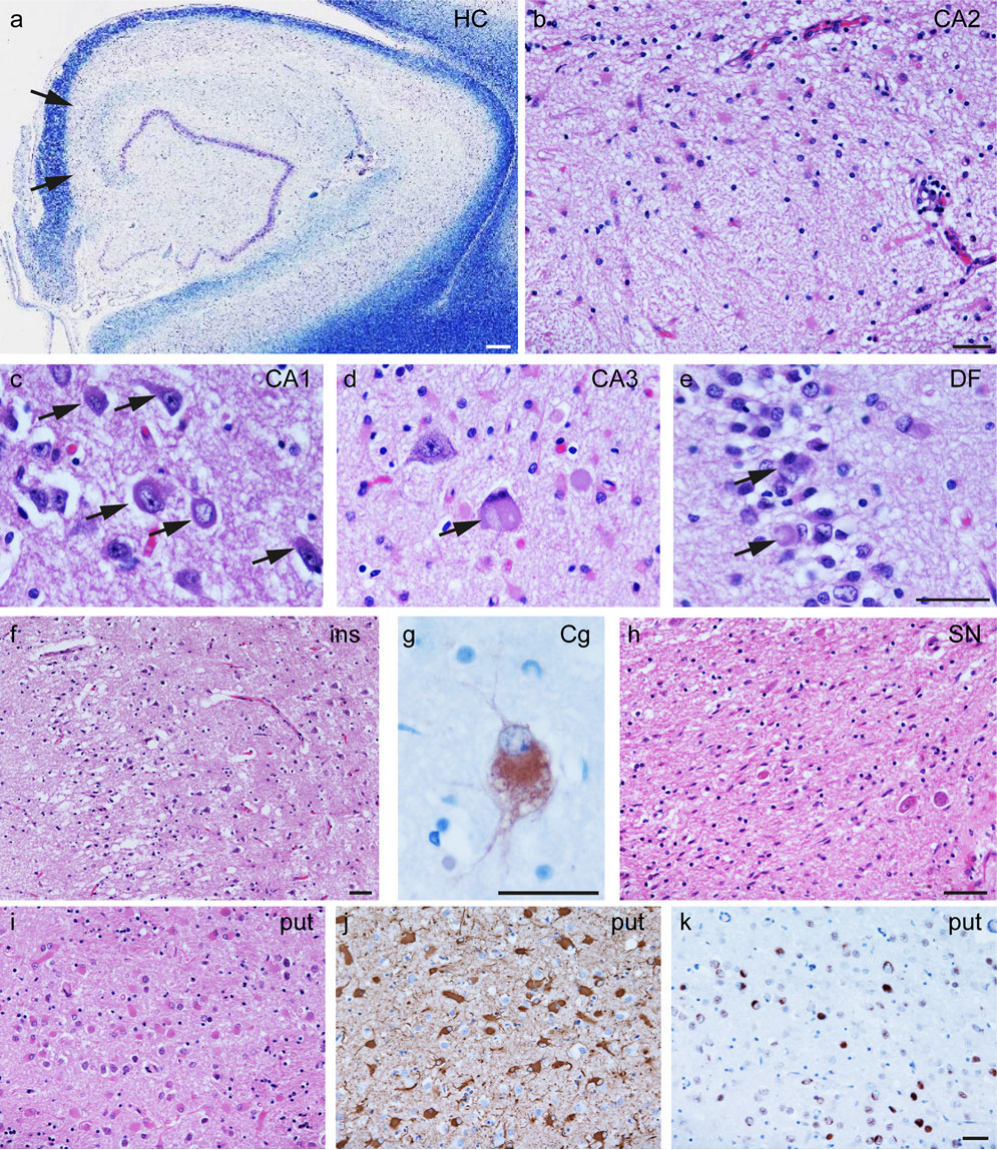
Histological findings. Representative images from the hippocampus (**a**–**e**), insular cortex (*ins*, **f**), cingulate gyrus (*Cg*, **g**), substantia nigra (*SN*, **h**) and putamen (*put*, **i**–**k**). In the hippocampus, there was severe neuronal loss in the CA2 and CA3 (**a**, *arrows*). The CA2 showed few residual neurons (**b**). Neuronal inclusions (*arrows*) with varying morphology are illustrated in the CA1 (**c**), CA3 (**d**) and the dentate fascia (*DF*, **e**). Superficial laminae of the neocortex showed marked neuronal loss with microvacuolation illustrated in the insular cortex (*ins*, **f**). Ballooned neurons were most frequent in the cingulate gyrus (*Cg*, **g**). The substantia nigra (*SN*) shows severe loss of pigmented neurons accompanied by gliosis (**h**). Abundant eosinophilic reactive astrocytes are visualised in the putamen (*put*, **i**) and confirmed by GFAP immunohistochemistry (**j**). TDP-43 immunoreactive inclusions were also detected in the putamen (**k**). Luxol fast blue (**a**), haematoxylin and eosin (*H&E*) (**b**–**f**, **h**, **i**), αB-crystallin (**g**), GFAP (**j**) TDP-43 (**k**). *Scale bar* 150 μm (**a**), scale bars in **b**, **e**–**h**, **k** represent 50 μm (**c**–**e** are at the same magnification, as are **i**–**k**)

**Fig. 5:**
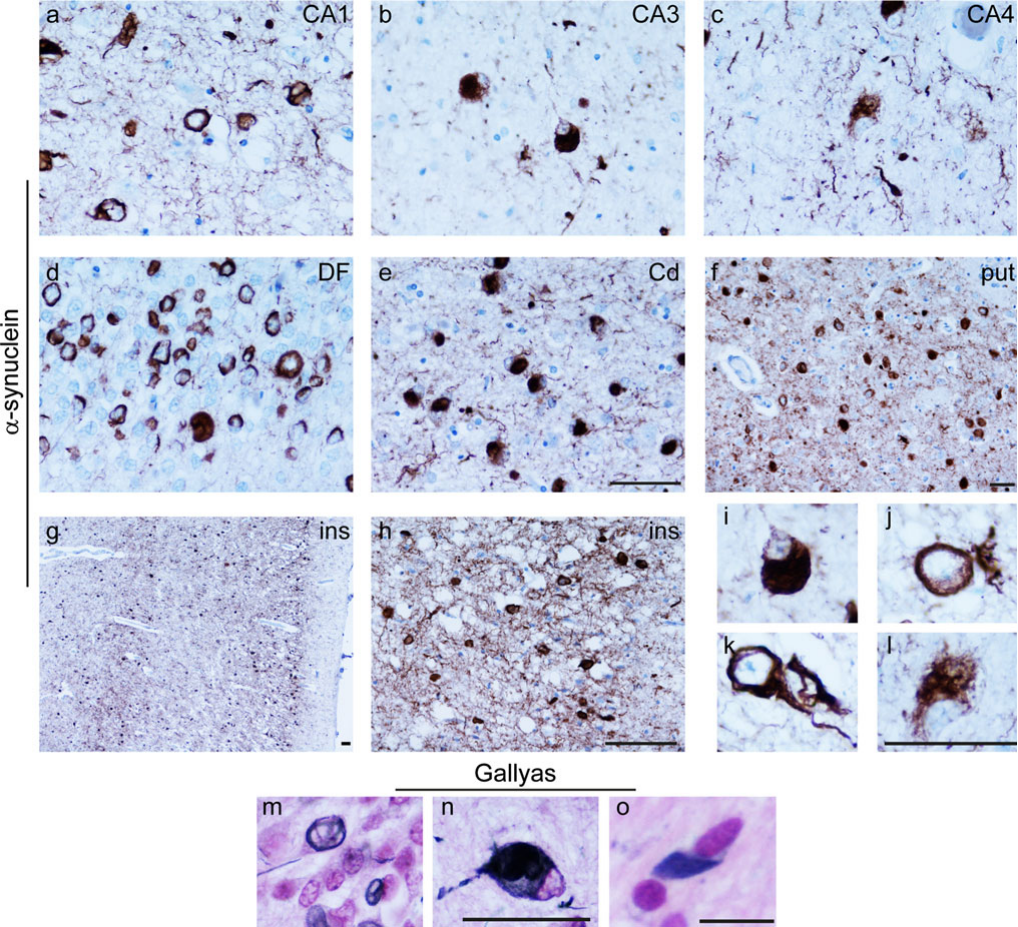
Characterisation of neuronal and glial α-synuclein inclusions. Thread-like α-synuclein immunoreactivity was observed to be widespread, shown here within the hippocampal regions CA1, CA3 and CA4 (**a**–**c**), caudate nucleus (*Cd*, **e**), putamen (*put*, **f**) and insular cortex (*ins*, **g**, **h**). Neuronal cytoplasmic inclusions immunoreactive for α-synuclein had a number of different morphological appearances globular (**i**), annular (**j**), neurofibrillary tangle-like (**k**) and diffuse (**l**). Gallyas silver impregnation demonstrated the presence of fibrillar protein in neuronal inclusions (*DF*, *m* and *CA4*, **n**) and also in GCI-like inclusions (pontine base, **o**). *Scale bars* in **e**–**h**, **l** represent 50 μm (**a**–**n**) (**a**–**e** are at the same magnification as are **i**–**l**, **m** and **n**). *Scale bar* in **o** represents 10 μm

Histological findings are summarised in Table 1 and illustrated in Figs. 4 and 5. The neocortical regions showed marked superficial vacuolation with neuronal loss and gliosis in all regions except the occipital cortex. Cortical neuronal depletion was most severe in the anterior temporal, piriform and insular cortices. In the hippocampal formation, neuronal loss affected all regions except the dentate fascia (DF) and this was most striking and severe in the CA2 and CA3 subregions where there was almost complete neuronal loss with accompanying gliosis. Mild neuronal loss accompanied by severe and uniformly distributed gliosis was observed in the caudate and putamen (Fig. 4i, j). There were virtually no remaining pigmented neurons in the substantia nigra pars compacta (Fig. 4h), and there was severe loss of neurons in the locus coeruleus and the dorsal motor nucleus of the vagus (DMV). No classical intraneuronal LBs were seen in the substantia nigra, locus coeruleus or DMV in H&E stained sections, likely due to the very severe neuronal loss. Cerebellar Purkinje cells were moderately depleted while the dentate nucleus was well preserved. In the rostral cervical spinal cord, the anterior horn motor neurons were preserved. Weakly basophilic NCIs with varied morphology, including well-defined round or oval structures and perinuclear annular or crescent shapes, were widespread and were prominent in the hippocampus, neocortex and striatum (Fig. 4a–k). Balloon neurons, best identified by αB-crystallin immunohistochemistry, were present in the frontal and temporal cortices and were most numerous in the cingulate gyrus (Fig. 4g).

**Table 1:** Semi-quantitative assessment and regional distribution of neuronal loss and α-synuclein pathology

	Neuronal loss	Neuronal α-synuclein pathology				α-Synuclein threads	Oligodendroglial α-synuclein inclusions
		Annular or crescent	Globular	Diffuse	NFT-like		
Cortex							
Frontal	+	++	+	+	–	++	+
Motor	+	++	+	+	–	+++	+
Temporal	+++	+++	+	+	–	+++	–
Parietal	+	+++	++	+	–	+++	–
Occipital	–	+	–	–	–	–	–
Cingulate	++	+++	++	+	–	+++	–
Insular	+++	+++	+	+	–	+++	–
Sub-cortical white matter							
Frontal	N/A	N/A	N/A	N/A	N/A	+	+
Motor	N/A	N/A	N/A	N/A	N/A	+	++
Temporal	N/A	N/A	N/A	N/A	N/A	+	+
Parietal	N/A	N/A	N/A	N/A	N/A	+	+
Occipital	N/A	N/A	N/A	N/A	N/A	+	–
Cingulate	N/A	N/A	N/A	N/A	N/A	+	+
Internal capsule	N/A	N/A	N/A	N/A	N/A	++	+
External capsule	N/A	N/A	N/A	N/A	N/A	++	+
Amygdala	+++	++	++	+	–	+++	+
Hippocampus							
DF	–	+++	+	+	–	++	–
CA4	++	++	+	+	+	+++	+
CA3	+++	+	+	–	–	++	–
CA2	+++	–	–	–	–	++	–
CA1	+	+++	+	+	+	+++	–
Subiculum	+	++	++	+	+	+++	–
Entorhinal cortex	++	+++	+	+	+	+++	–
Transentorhinal cortex	++	++	+	+	+	++	–
Caudate	+	+	+++	++	+	+++	–
Putamen	+	++	+++	++	+	+++	–
Globus pallidus	–	+	–	–	–	+	+
Thalamus	–	–	+	+	–	+	+
Subthalamic nucleus	–	–	–	+	–	++	–
Red nucleus	–	–	–	–	–	+	+
III nerve nucleus	–	+	+	+	–	+	–
Substantia nigra	+++	–	–	–	–	++	+
Locus coeruleus	+++	–	–	–	–	++	–
Pontine nuclei	–	–	–	+	–	+	+
Pontine base white matter	N/A	–	–	–	–	++	++
Dorsal motor nucleus of vagus	+++	–	–	–	–	++	–
Twelfth nerve nucleus	–	–	–	–	–	+	–
Inferior olive	+	–	–	++	–	+	–
Cerebellar hemisphere Purkinje cells	++	–	–	–	–	–	N/A
Cerebellar hemisphere white matter	N/A	N/A	N/A	N/A	N/A	++	++
Dentate nucleus	–	–	–	–	–	–	–
Cervical cord anterior horn	–	–	–	–	–	–	–
Cervical cord white matter	N/A	N/A	N/A	N/A	N/A	+	–

Oligodendroglial α-synuclein: cytoplasmic inclusions usually with similar morphology to GCIs of MSA, less frequently resembling coiled bodies

*N/A* not applicable, *NFT* neurofibrillary tangle

### Distribution and morphology of α-synuclein immunoreactive inclusions

Immunohistochemical staining for α-synuclein demonstrated widespread neuronal and glial inclusions (Table 1; Fig. 5). Amongst the areas most severely affected by neuronal α-synuclein pathology was the hippocampus (Fig. 5a–d), in which the DF and CA1 contained many inclusions. Inclusions were sparse in the CA2 and CA3 subregions reflecting the paucity of residual neurons in these areas. The caudate and putamen contained a remarkable load of neuronal α-synuclein-positive inclusions affecting all regions of each nucleus to a similar extent (Fig. 5e, f). In the neocortex, there was a distinct pattern in which the superficial and deep cortical laminae were most severely affected (Fig. 5g, h). NCIs had variable morphology; many were clearly defined globular inclusions resembling LBs (Fig. 5i), while others were annular or crescent shaped situated around the nucleus (Fig. 5j), less frequently they resembled neurofibrillary tangles (Fig. 5k). Small numbers of neurons showed a diffuse finely granular pattern of α-synuclein immunoreactivity (Fig. 5l). In most areas, neuronal inclusions were accompanied by threads of varying width, up to 10 μm, representing α-synuclein accumulation in cell processes. In cross section, these appeared as dots and round–oval structures. The argyrophilic nature of fibrillar α-synuclein inclusions was demonstrated using Gallyas silver impregnation (Fig. 5m–o).

### Glial inclusions

**Fig. 6:**
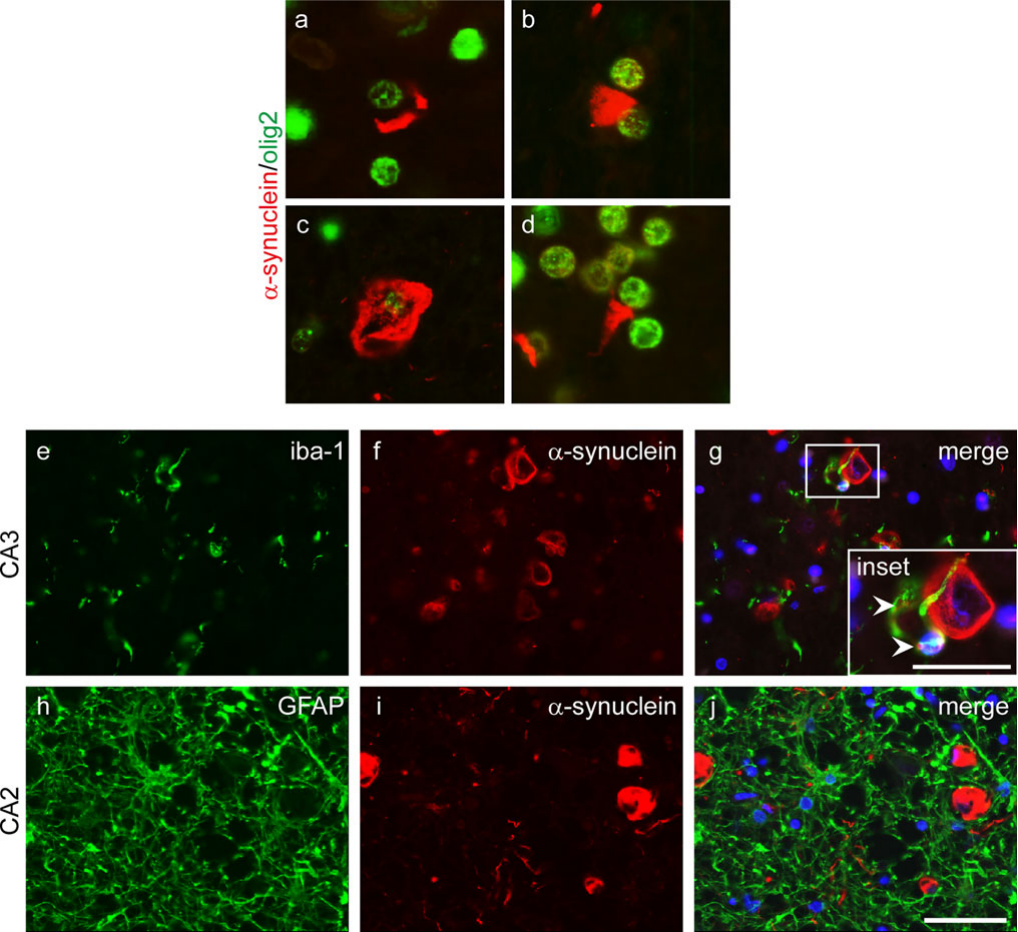
Glial inclusions. Representative double immunofluorescence images probed with α-synuclein (*red*) and the oligodendroglial marker, olig2 (*green*). Composite merged images show α-synuclein immunoreactive inclusions in oligodendrocytes resembling GCIs in the white matter of the frontal cortex (**a**, **d**), the pons (**b**) and the alveus of the hippocampus (**c**). Close proximity is observed between a subset of activated microglia, as detected by iba-1 (*green*) and α-synuclein (*red*) inclusion containing neurons, this occasionally involved encircling of α-synuclein-containing neurons by iba-1-positive processes (**e**–**g**). A small proportion of these microglia contained α-synuclein immunoreactivity (**g**, *inset* *arrowheads*). A high level of reactive astrogliosis was detected throughout all brain regions examined, without evidence of α-synuclein expression within astrocytes or their processes (**h**–**j**). DAPI nuclear stain (*blue*). *Scale bar* in **j** represents 50 μm (**a**–**j**). *Scale bar* in **g** *inset* represents 25 μm

A significant numbers of glial, α-synuclein-positive inclusions were observed, many of which resembled the GCIs of MSA (Fig. 6a–d) and thus are hereafter referred to as GCI-like, while others had appearances similar to coiled bodies (CBs) as previously described in PD [81]. The distribution of GCI-like pathology also resembled MSA as they were most frequent in the pontine base, cerebellar white matter and in the white matter underlying the motor cortex (Table 1). They were also immunoreactive for recognised GCI markers including αB-crystallin [63]. Double immunofluorescence of α-synuclein with the oligodendroglial marker olig-2 confirmed that GCI-like inclusions were in oligodendrocytes (Fig. 6a–d).

The relationship between microglia and astrocytes with α-synuclein pathology was also investigated. A subset of microglia, detected using the microglial marker iba-1, was observed in close proximity to, in some cases with processes encircling, α-synuclein inclusion containing neurons (Fig. 6e–g). A small number of microglia investigated in both the hippocampus and neocortex contained thread-like α-synuclein immunoreactivity (Fig. 6g inset, arrows). Marked reactive astrogliosis, detected by GFAP immunofluorescence, was observed, however, double immunofluorescence studies showed no α-synuclein immunoreactivity in astrocytic processes in any regions including the severely affected CA2, where there was a high density of α-synuclein-positive threads (Fig. 6h–j).

### Tau expression within neuronal inclusions and co-localisation with α-synuclein

**Fig. 7:**
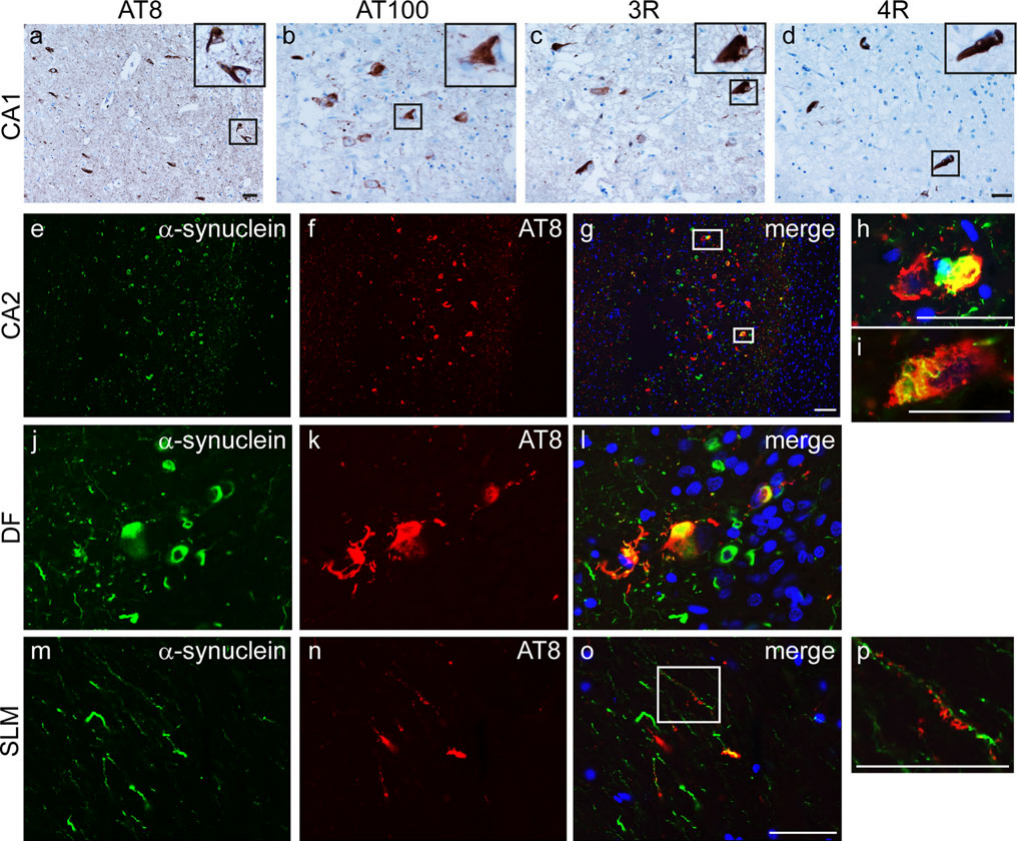
Tau expression and co-localisation with α-synuclein. Representative images from CA1 probed using immunohistochemistry with phospho-tau antibodies: AT8 (**a**) and AT100 (**b**). Inclusions contained a mixture of 3-repeat (**c**) and 4-repeat (**d**) tau isoforms. Double immunofluorescence images of CA2 (**e**–**i**) and DF (**j**–**l**) probed for α-synuclein (*green*) and AT8 (*red*) show strong co-localisation in a subset of neurons. AT8 immunoreactivity is detected on the dendritic processes of DF granule cells which express α-synuclein in the stratum lacunosum-moleculare (*SLM*, **m**–**p**). DAPI nuclear stain (blue). *Scale bars* represent 50 μm (**b**–**d** are at the same magnification as are **e**–**g**, **j**–**o**)

Phosphorylated tau was identified using immunohistochemical staining for AT8, recognising Ser202 and Thr205 (Fig. 7a). This demonstrated granular pre-tangles, neurofibrillary tangles and neuropil threads in the hippocampal formation, most frequent in the CA1, CA4 and subiculum. They were also present in moderate numbers in the entorhinal and transentorhinal cortices and DF (shown in the DF, Online resource 2). There was no neocortical tau pathology and only rare neuropil threads were found in the midbrain and pontine tegmenta. Overall the tau pathology conformed largely to Braak and Braak stage II Alzheimer-type pathology, although involvement of the DF at this stage would not be expected [6]. Further support for Alzheimer-type tau deposition was provided using the AT100 antibody, recognising tau phosphorylated at Thr212 and Ser214 with paired helical filament conformation, which labelled a proportion of neurofibrillary tangles and by isoform specific antibodies, which showed a mixture of 3- and 4-repeat tau isoforms (Fig. 7b–d).

Double immunofluorescence microscopy was used to investigate the relationship between α-synuclein and tau. This showed that, particularly in the CA1 and DF, α-synuclein and tau co-localised in a proportion of inclusions (Fig. 7e–l). In addition, AT8 immunoreactivity was detected on the dendritic processes of DF neurons in the stratum lacunosum-moleculare (SLM), which also expressed α-synuclein (Fig. 7m–p).

### α-Synuclein within inclusions is phosphorylated and inclusions are immunoreactive for ubiquitin and p62

**Fig. 8:**
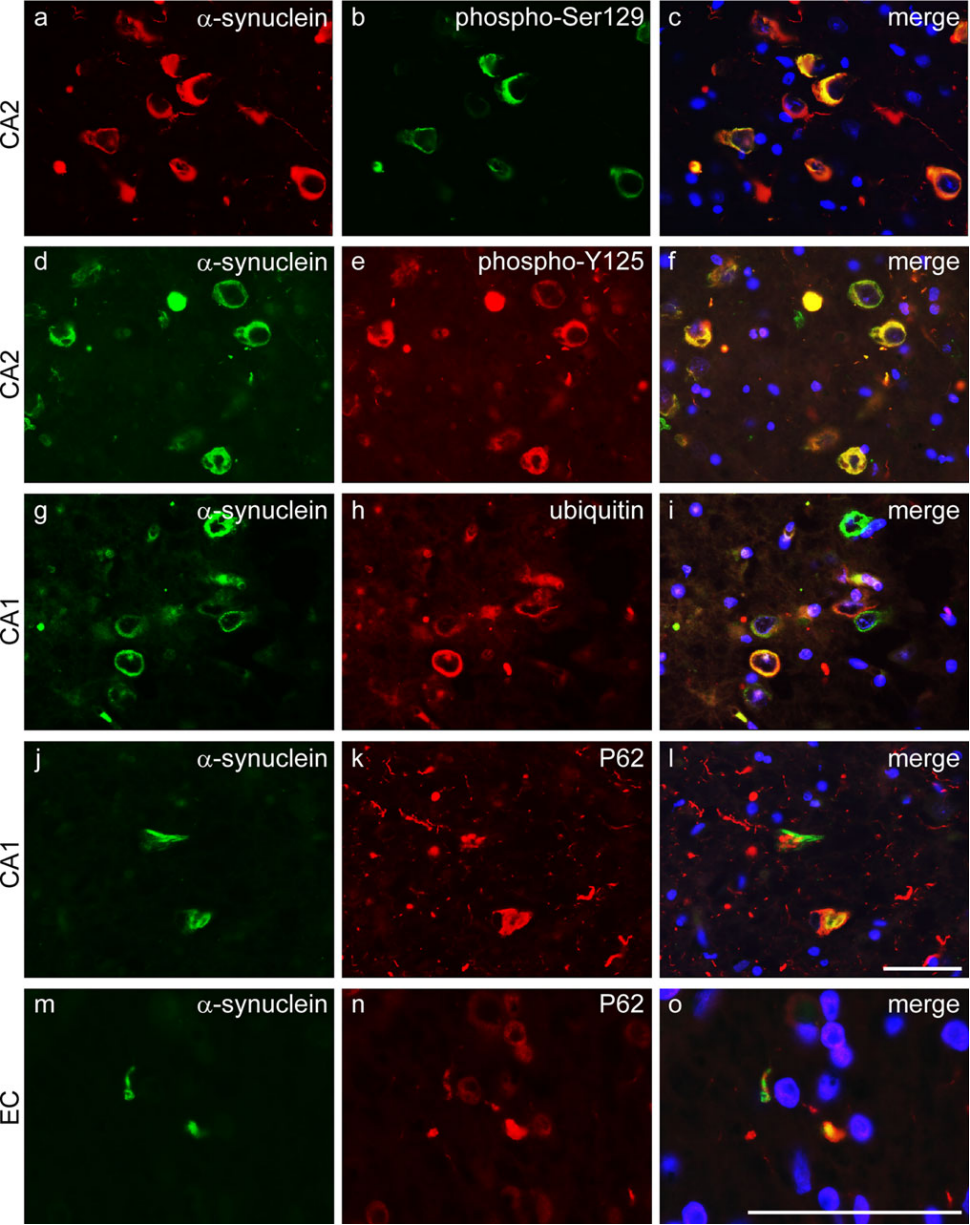
α-Synuclein is phosphorylated and inclusions contain ubiquitin and p62. Representative images of the CA2 region of the hippocampus probed by double immunofluorescence with total α-synuclein (*red*) and α-synuclein phospho-S129 (*green*) (**a**–**c**), or α-synuclein (*green*) and α-synuclein phospho-Y125 (*red*) (**d**–**f**) show near complete co-localisation indicating that α-synuclein is phosphorylated at both epitopes. The majority of α-synuclein immunoreactivity (*green*) within neuronal inclusions co-localised with ubiquitin (*red*), shown in CA1 neurons (**g**–**i**). Many α-synuclein-positive (*green*) neuronal inclusions also contained P62 (*red*) (**j**–**l**) and a similar pattern was observed in GCI-like inclusions illustrated in the white matter underlying the entorhinal cortex (*EC*, **m**–**o**). DAPI nuclear stain (*blue*). *Scale bars* represent 50 μm (**a**–**l** are at the same magnification as are **m**–**o**)

α-Synuclein in LBs is widely reported to be extensively phosphorylated [23, 82]. Double immunofluorescence staining for total α-synuclein and α-synuclein phospho-Ser129 (Fig. 8a–c) or phospho-Y125 (Fig. 8d–f) showed almost complete overlap of immunoreactivity within inclusions, implying the near total phosphorylation of α-synuclein at both epitopes. Ubiquitin and p62 co-localised with the majority of neuronal α-synuclein-positive inclusions (Fig. 8g–l) and also GCI-like inclusions (Fig. 8m–o).

### Additional pathological findings

TDP-43 immunohistochemistry identified moderate numbers of NCIs in the caudate and putamen (Fig. 4k), while these were sparse in the amygdala, DF, CA4, CA1, subiculum, entorhinal cortex and transentorhinal cortex. A single neuronal nuclear TDP-43 immunoreactive inclusion was noted in the entorhinal cortex. Aβ deposition was not detected.

## Discussion

We provide detailed clinical, genetic and neuropathological characterisation of an α-synucleinopathy in a patient carrying a G51D α-synuclein mutation, clinically diagnosed with juvenile parkinsonism. Neuropathological analysis revealed a combination of the characteristic profile of SN and LC neuronal loss together with neuronal α-synuclein immunoreactive inclusions consistent with PD as well as severe hippocampal, cortical and striatal α-synuclein pathology. An additional feature was the presence of GCI-like oligodendroglial inclusions with a distribution similar to that found in MSA. This case shows some similarity to reports of A53T mutation and multiplication of *SNCA* (Table 2) and also to a Japanese kindred reported in an abstract as carrying a G51D *SNCA* mutation, but without detailed segregation data [50, 51]. However, features including dense accumulation of α-synuclein-positive inclusions in the striatum and very severe neocortical α-synuclein pathology affecting both superficial and deep cortical laminae distinguish this case from other reported cases with *SNCA* mutations.

**Table 2:** Neuropathology of α-synuclein mutation cases where α-synuclein immunohistochemistry data are available

	Mutation	Number of cases (*n*)	Neuronal loss				α-Synuclein pathology				Tau Braak and Braak stage	TDP-43 pathology
			SN	LC	CA1	CA2/3	Pattern	Striatum	Neocortical distribution	Glial inclusions		
Golbe et al. [26] and Duda et al. [19]	A53T	2	Severe	Mild–moderate	Yes	No	PD-like	Moderate grains and threads	Deep laminae	Not described	I	Not described
Spira et al. [77]	A53T	2	Severe	Severe	No	Severe	PD-like	None	Deep laminae	None	0	Not described
Markopoulou et al. [48]	A53T	2	Severe	Mild	Severe (*n* = 1)	CA2 (*n* = 1)	PD-like	Grains and threads (*n* = 1)	Deep laminae	Small numbers GCIs	IV (*n* = 1), I (*n* = 1)	Limbic and cortical (*n* = 1, with CA1 neuronal loss)
Seidel et al. [72]	A30P	1	Severe	Not described	Not described	Not described	PD-like	LBs	Deep laminae	Small numbers CB and astrocytes of PD type	II	Not described
Zarranz et al. [84]	E46 K	1	Moderate–severe	Mild	Not described	Not described	PD-like	None	Present, distribution not described	Not described	0	Not described
Obi et al. [57]	Duplication	1	Severe	Severe	None	Moderate	Features of PD and MSA	None	Present, distribution not described	Small numbers GCIs and CB	I	Not described
Ikeuchi et al. [32]	Duplication	1	Severe	Not described	None	Severe	PD-like	Not described	Present, distribution not described	Few, type not specified	III	Not described
Gwinn-Hardy et al. [27]	Triplication	1	Severe	Severe	None	Severe	Features of PD and MSA	LBs and neurites	Predominantly deep laminae	Small numbers GCIs and CB	0	Not described
Farrer et al. [21]	Triplication	1	Severe	Severe	None	Severe	PD-like^a^	Not described	Present, distribution not described	Not described	Not described	Not described
This report	G51D	1	Severe	Severe	Mild	Severe	Features of PD and MSA	Frequent neuronal inclusions and threads	Superficial and deep laminae	GCIs and CB	II	Limbic

*SN* substantia nigra, *LC* locus coeruleus, *N/A* not available, *PD* Parkinson’s disease, *CB* coiled bodies

^a^Detailed description of α-synuclein pathology not provided

In comparison with other reported mutations in the *SNCA* gene, the age of onset in this family, clinical features and progression are most similar to the *SNCA* triplication [16] and A53T mutations, which are typically associated with young-onset PD frequently associated with cognitive impairment and hallucinations. This is similar to the family where the proband presented early at 19 years while his father and sister presented at age 39 and 40 years, respectively.

In this family, an *SNCA* G > A heterozygous mutation at codon 51 causes a glycine to aspartic acid amino acid change (Fig. 1a), which segregates with the disease (Fig. 1b) and was not found in over 4,500 control individuals. The G51D mutation is located in the N-terminal domain of the protein, a region required for lipid affinity and membrane binding and may thus influence these functions (Fig. 2) [20, 37, 68]. As the A53T and E46K mutations result in faster fibrillisation of α-synuclein [13, 62], we postulate that the G51D mutation might have a similar effect.

The neuropathological findings included some features of both PD and MSA. In common with PD and MSA, there was severe neuronal loss in the SN. Neuronal α-synuclein pathology had a distribution compatible with PD in that brainstem, limbic and cortical regions were affected. Unusual for PD, however, was the severe neuronal loss in the CA2/3 subregions of the hippocampus and the extensive accumulation of neuronal α-synuclein in the hippocampus, including the DF, and striatum [7, 8, 38, 52]. Involvement of the DF by neuronal α-synuclein inclusions has been described previously in the case of DLB with additional MSA-type pathology and in some cases of MSA [73, 74, 78]. The severe neuronal loss observed in CA2/3 of this case bears similarity to that described in association with A53T *SNCA* mutation [48, 77] and in cases of multiplication of *SNCA*, though this pattern of loss was suggested to be a unique feature of the latter [21] (Table 2). As frequently observed in PD and DLB, and also reported in conjunction with A53T *SNCA* mutation, α-synuclein-positive threads were frequent in CA2/3 [17, 18, 34, 35]. The severe neuronal loss we observed is likely to be associated with the susceptibility of these hippocampal subregions to α-synuclein accumulation.

Oligodendroglial pathology in the form of GCI-like inclusions in regions such as the posterior frontal white matter, pontine base and cerebellar white matter was also notable and prompts comparison with MSA. In common with GCIs in MSA, these inclusions were immunoreactive for αB-crystallin [63]. Oligodendroglial pathology with similar morphological appearances has been described in cases with *SNCA* multiplication or A53T mutation (Table 2) and, interestingly, was also reported in a sporadic DLB case [74]. Neuronal and glial α-synuclein pathology in the striatum is also a common feature of MSA and we noted very frequent striatal neuronal α-synuclein immunoreactive inclusions coupled with severe gliosis. However, in notable contrast to MSA, in which the putamen has a gradient of pathology most severely affecting the posterior and dorsal aspects of this nucleus with less severe involvement of the caudate, there was a uniform distribution of pathology in all regions of both nuclei in this case [1, 58]. This pattern of striatal pathology appears to be unique to our case as it has not been described in the context of other *SNCA* mutations (Table 2).

Hippocampal sclerosis with severe neuronal loss affecting CA1 and the subiculum is a feature of many neurodegenerative diseases and is often associated with TDP-43 immunoreactive inclusions in residual neurons in these regions and elsewhere [64]. TDP-43 pathology is reported to be frequent in DLB cases, but is considerably rarer in MSA and in PD [3, 24, 30, 53]. While we observed sparse TDP-43-positive inclusions in these areas, the overall pattern of neuronal loss, being most severe in CA2/3, did not resemble typical hippocampal sclerosis. Of particular note was the abundance of TDP-43 immunoreactive NCIs in the caudate and putamen, regions also vulnerable to TDP-43 pathology in FTLD-TDP [9]. We did not observe TDP-43-positive inclusions in neocortex or brainstem motor nuclei. A further neuropathological feature, which may be distinctive in the G51D α-synuclein mutation, is the distribution of neocortical α-synuclein pathology. In PD, MSA and other *SNCA* mutations, α-synuclein pathology is predominantly found in the deep cortical laminae (Table 2) [19, 26, 27, 48, 72, 77] compared with our observation of severe involvement of both the superficial and deep cortical layers.

We further investigated the nature of intracellular inclusions and Gallyas silver impregnation indicated the presence of fibrillar protein. Phosphorylation of α-synuclein at Ser129 is a feature of LB pathology and may promote oligomerisation, while phosphorylation at Y125 may increase protein fibrillisation of α-synuclein [11, 29, 54]. Using immunofluorescence, we showed phosphorylation of α-synuclein at both Y125 and Ser129. Although the majority of neuronal and glial inclusions were immunoreactive for p62 and ubiquitin, a proportion remained unstained and these possibly represented an early phase of inclusion formation [40, 41, 44]. A varying degree of tau pathology has been reported in cases of *SNCA* mutation or multiplication sometimes co-localising with α-synuclein in neuronal inclusions [19, 27, 32, 48, 57, 72, 84]. We report tau pathology in the hippocampal formation and entorhinal cortex corresponding to Braak and Braak stage II, but with additional involvement of the DF. Phosphorylated tau co-localised with a subpopulation of neuronal α-synuclein inclusions, particularly in the CA1 and DF. A relationship between tau and both the levels and aggregation state of α-synuclein, such that greater numbers of tau-positive inclusions may increase α-synuclein pathology, is well described [14, 15, 25, 46]. More recently, it has been shown that α-synuclein oligomers can seed tau aggregation in vitro and this may explain the occurrence of tau in a proportion of α-synuclein-containing inclusions [45].

The defining neuropathological hallmark of MSA is the presence of α-synuclein-containing GCIs coupled with neurodegeneration in the striatonigral and/or olivopontocerebellar regions [79]. The mechanism of GCI formation is currently unknown and mature oligodendrocytes are not thought to express α-synuclein under normal circumstances [76]. Evidence of GCI-like pathology in cases of *SNCA* multiplication [27, 57], A53T mutation [48] and in this case of G51D mutation, provide a strong link between these mutations and the pathological mechanisms of MSA. One of the earliest stages of MSA pathogenesis may involve the overexpression or aberrant localisation of α-synuclein in oligodendrocytes, where it becomes fibrillar [69] and forms GCIs [39]. Greater understanding of the effect of the G51D and A53T mutations and *SNCA* multiplication may shed further light on the pathological cascades, which result in GCI formation. The data presented indicate that G51D *SNCA* mutation results in a neuropathological profile, which shares some neuropathological features of both PD and MSA and, therefore understanding the consequences of this mutation, has the potential to provide greater insight into the role of α-synuclein mutation or dysfunction in the pathogenesis of PD and also MSA. Understanding the biology of this G51D *SNCA* mutation could help us to target pathways in PD, MSA and other synucleinopathies, which lead to neuronal and glial α-synuclein accumulation.

## Electronic supplementary material

Below is the link to the electronic supplementary material.

Online resource 2. Supplementary Fig. 1 Phospho-tau immunoreactivity is detected in granule cells of the DF. Representative images from the DF of immunohistochemical staining for phospho-tau (AT8, a), α-synuclein (b) ubiquitin (c) and p62 (d) demonstrating that tau is present in a minority of neuronal inclusions. Scale bar in d represents 50 μm in a–d.

Supplementary material 1 (DOCX 16 kb)

Supplementary material 2 (TIFF 1430 kb)

## Abbreviations

Aβ: Amyloid beta
ALS: Amyotrophic lateral sclerosis
CA: Cornu ammonis
CB: Coiled bodies
Cg: Cingulate gyrus
CN: Caudate nucleus
DF: Dentate fascia
DLB: Dementia with Lewy bodies
DMV: Dorsal motor nucleus of the vagus
FTLD: Frontotemporal lobar degeneration
GCI: Glial cytoplasmic inclusions
LB: Lewy bodies
LN: Lewy neurites
MSA: Multiple system atrophy
NAC: Non-amyloid-β-component
NCI: Neuronal cytoplasmic inclusions
NNI: Neuronal nuclear inclusions
PBs: Pale bodies
PNs: Pale neurites
PD: Parkinson’s disease
Pt: Putamen
SLM: Stratum lacunosum-moleculare
SN: Substantia nigra
TAR-DNA-binding protein-43: TDP-43
WT: Wild type
