# Supplementary material for: α-Synucleinopathy associated with G51D SNCA mutation: a link between Parkinson’s disease and multiple system atrophy?
Source: Acta Neuropathol. 2013 Feb 12;125(5):753–69. doi: 10.1007/s00401-013-1096-7 (PMC3681325; doi:10.1007/s00401-013-1096-7)
Supplement: Supplementary file 1 — Supplementary material 1 (DOCX 16 kb) [file 401_2013_1096_MOESM1_ESM.docx]

**Supplementary table 1. Primary antibodies used in the study**

| *Antibody (clone/epitope)* | *Clonality* | *Supplier (catalogue number)* | *Application* | *Dilution* | |
| --- | --- | --- | --- | --- | --- |
| α−synuclein (KM51) | monoclonal | Vector (VP-A106) | IHC/IF | 1:50 | |
| α−synuclein (amino acids 111-131, C-terminal) | polyclonal | Abcam (ab15530) | IF | 1:800 |  |
| Phospho-α-synuclein (Ser129) | polyclonal | Abcam (ab59264) | IF | 1:100 |  |
| Phospho-α-synuclein (Y125) | polyclonal | Abcam (ab10789) | IF | 1:75 |  |
| AT100 (Thr212/Ser214) | monoclonal | Innogenetics (#90337) | IHC | 1:100 |  |
| Aβ (6F/3D) | monoclonal | DAKO (M0872) | IHC | 1:100 |  |
| AT8 (Ser202/Thr205) | monoclonal | Source Bioscience (90206) | IHC/IF | 1:600 |  |
| α−B-Crystallin (G2JF) | monoclonal | Novocastra (ABCRYS-512) | IHC | 1:300 |  |
| 3R | monoclonal | Courtesy of Dr. Rohan De Silva | IHC | 1:100 |  |
| 4R | monoclonal | Courtesy of Dr. Rohan De Silva | IHC | 1:100 |  |
| Ubiquitin | polyclonal | DAKO (Z0458) | IHC | 1:200 |  |
| P62 (3/P62 LCK LIGAND) | monoclonal | BD Transduction (610833) | IHC/IF | 1:100 |  |
| TDP-43 (2E2-D3) | monoclonal | Abnova (H00023435-M01) | IHC | 1:800 |  |
| CD68 (EBM11) | monoclonal | DAKO (M0718) | IHC | 1:150 |  |
| α−Internexin | polyclonal | Abcam (967654) | IHC | 1:75 |  |
| GFAP | polyclonal | Dako (Z0334) | IHC/IF | 1:1000 |  |
| Iba-1 | polyclonal | Wako (091-19741) | IF | 1:500 |  |
| Olig-2 | polyclonal | Abcam (ab42453) | IF | 1:500 | |
|  |  |  |  |  | |

IHC, immunohistochemistry; IF, Immunofluorescence
