# Supplementary figures and images for: α-Synucleinopathy associated with G51D SNCA mutation: a link between Parkinson’s disease and multiple system atrophy?
Source: Acta Neuropathol. 2013 Feb 12;125(5):753–69. doi: 10.1007/s00401-013-1096-7 (PMC3681325; doi:10.1007/s00401-013-1096-7)

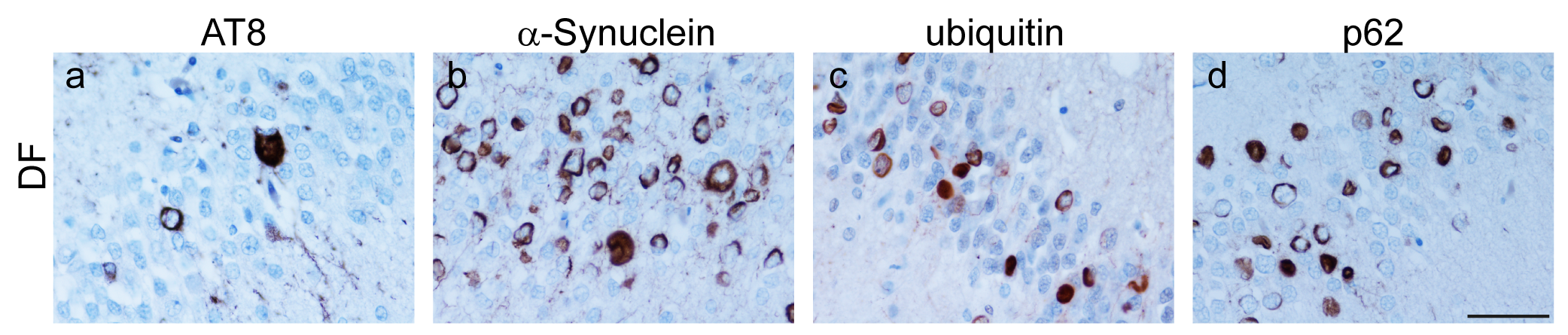

Supplement: Supplementary file 2 — Supplementary material 2 (TIFF 1430 kb) [file 401_2013_1096_MOESM2_ESM.tif]
